# Puberty-Onset Selective Mutism in a Female Adolescent With Autism Spectrum Disorder

**DOI:** 10.7759/cureus.82280

**Published:** 2025-04-15

**Authors:** Tiago Soares, Joana Coelho Santos, Daniela Couto

**Affiliations:** 1 Department of Child and Adolescent Psychiatry, Local Health Unit of Western Lisbon, Lisbon, PRT

**Keywords:** autism spectrum disorder, child and adolescent psychiatry, cognitive behavioral therapy, pregabalin, puberty onset, school refusal, selective mutism, severe anxiety

## Abstract

Selective mutism (SM) is a rare anxiety disorder typically diagnosed in early childhood. It is characterized by a persistent failure to speak in specific social situations despite having the ability to verbalize in others. Although the onset of SM during adolescence is uncommon, its emergence during puberty or major life transitions may reflect an atypical form of anxiety that is more frequently observed in individuals with autism spectrum disorder (ASD). We describe a case of an 11-year-old girl who developed SM following the onset of puberty and significant environmental changes, including school transition and relocation. She was subsequently diagnosed with ASD. A multidisciplinary treatment approach involving cognitive behavioral therapy, fluoxetine, and pregabalin led to complete remission of SM and marked improvements in academic and social functioning. This case underscores the importance of recognising atypical anxiety presentations in adolescents with ASD and highlights the value of early, individualized, and multimodal interventions. It also raises ethical considerations regarding the temporary use of covert medication in cases with severe resistance to treatment.

## Introduction

Selective mutism (SM) is an anxiety disorder defined by a persistent inability to speak in certain social settings despite having the capacity to do so in others, often interfering with academic or social development [1]. It typically emerges before the age of five years, although diagnosis is often delayed until school entry [2]. Its prevalence is estimated between 0.03% and 1% of the population [3]. SM frequently co-occurs with other anxiety disorders, particularly social anxiety disorder [2], and is also associated with neurodevelopmental conditions such as autism spectrum disorder (ASD) [4].

ASD is characterized by persistent deficits in social communication and interaction, along with restricted and repetitive behaviors [1]. Up to 50% of individuals with ASD experience clinically significant anxiety, which may manifest in atypical ways such as intense social withdrawal, nonverbal behaviors, or mutism [5]. In these cases, SM may not reflect a primary language impairment but rather an extreme expression of anxiety, sensory overstimulation, or social avoidance [5].

While SM most commonly arises in early childhood, its emergence or worsening during adolescence is considered unusual [6]. However, when SM appears during this developmental stage, particularly in the context of significant developmental or environmental changes, it may reflect an atypical presentation of anxiety more frequently seen in individuals with ASD [5-7]. The onset of puberty, accompanied by hormonal, cognitive, and psychosocial changes, can intensify existing vulnerabilities, and events such as changing schools or relocating may act as triggers for SM in susceptible individuals [2,7].

Despite increasing recognition of anxiety in adolescents with ASD, late-onset or reactivated SM in this group remains under-reported and poorly understood [2,4,8]. There is a notable lack of systematic research on adolescent-onset selective mutism in individuals with ASD, with most available literature limited to isolated case reports or small series [4,8]. Recognizing atypical anxiety presentations in this population is essential to enable timely intervention and prevent long-term functional impairment [5].

This case report contributes to the growing awareness of such presentations by describing an 11-year-old girl with ASD and severe anxiety who developed SM following puberty onset and major life changes. We explore her diagnostic workup, therapeutic intervention, and clinical outcome, including the successful use of adjunctive pregabalin and ethically guided covert medication in a treatment-resistant presentation.

## Case presentation

An 11-year-old Portuguese girl was referred to child and adolescent psychiatry outpatient services in August 2022 for a one-year history of severe anxiety symptoms, including selective mutism, school refusal, and social withdrawal. Her difficulties began in 2021, shortly after the COVID-19 lockdown, following a relocation to a new home within the same city and a transition to a larger public school. She expressed significant distress about leaving her previous home. She had anticipated attending the same school as her older brother but was unable to do so due to a lack of available spots. These environmental stressors coincided with the onset of puberty, marked by menarche at the age of 10 years.

Before moving, the patient had attended a small private school in Lisbon, where she was described as introverted yet capable of forming friendships and participating in class. After the transition, she became increasingly anxious, ceased verbal communication in all settings outside the home, including school and extended family, and withdrew socially. Within three months of enrollment, she stopped attending school entirely and failed the academic year, was not enrolled in extracurricular activities, and expressed no interest in joining any.

She lived with her mother, father, and 12-year-old brother. She continued to speak with them. Her routine involved watching cartoons for around three hours, playing a fantasy video game, and caring for several pets. Attempts to leave home provoked agitation and crying, though she would eventually comply when insisted, returning home calmly. She had no peer relationships, and previous friendships had largely been mediated through her brother.

A brief period of poor hygiene occurred over approximately two weeks, during which she refused to bathe. This behavior was promptly addressed through consistent parental support, including gentle prompting, establishing a predictable hygiene routine, and positive reinforcement. Her functioning at home was otherwise described as intact.

There were repeated attempts to have a consultation in child and adolescent psychiatry and psychology when she was 10 years old, but she persistently refused to attend appointments and never engaged in care. An occupational therapist also visited her home twice, but she consistently avoided interaction by retreating to her room.

Her early developmental history was largely unremarkable. She was born following an uncomplicated pregnancy and delivery to a 36-year-old mother and a 55-year-old father. The mother experienced postnatal blues and increased anxiety during maternity leave. The patient was enrolled in daycare at the age of three months and later began kindergarten at approximately five years of age. She was breastfed for two months before switching to a standard infant formula, with no reported feeding difficulties. She had delayed independent walking at 20 months due to joint hypermobility but achieved early language milestones on time. There was no known history of chronic physical illness, and general health was reported as satisfactory.

Since early childhood, the patient had exhibited an inhibited temperament, with strong separation anxiety, cautious observation, and low behavioral reactivity. She was described as easy to manage and relatively tolerant of frustration. She engaged in shared play with her brother but mostly in a repetitive way (e.g., aligning objects) with limited pretend play, reportedly only putting dolls to sleep. However, she demonstrated joint attention, imitation of parental behaviors, and interest in her environment.

From the age of eight years, she developed marked food selectivity, avoiding breakfast and refusing fruits, vegetables, or any green foods. Her diet was limited to rice, pasta, beef, eggs, and soup. She began multivitamin supplementation at the age of nine years. Sensory sensitivities, particularly to clothing textures and loud noises, had been long-standing and worsened after the move. She consistently refused to eat with her family, citing an aversion to food smells and the sound of chewing. She rigidly adhered to specific routines, including wearing only three outfits and maintaining a highly organized bedroom with fixed object placements.

Since the first grade, the patient attended a small private school in Lisbon through her primary years. During this time, she was described by teachers as academically capable, with age-appropriate reading and writing skills. Despite being socially reserved, she followed instructions, completed assignments, and demonstrated a solid understanding of academic content. There were no reported learning difficulties or concerns regarding attention or cognitive development. Her academic performance was considered satisfactory until the transition to the larger public school.

The family psychiatric history was significant for maternal panic disorder. A maternal uncle was described as having autistic traits, although never formally diagnosed. A paternal uncle had a diagnosis of delusional disorder, with two previous inpatient psychiatric admissions and treatment with long-acting antipsychotic injections. Her maternal grandfather had Alzheimer’s disease.

On mental state examination, the patient was mute, avoided eye contact, and exhibited stereotyped motor behaviors, including arm rubbing and rocking. Her posture and body language were consistently avoidant. She often walked glued to her mother's side, hiding her face behind long strands of hair as if using them as a physical shield.

The patient exhibited a complete absence of verbal or nonverbal interaction with the examiner during the assessment. Her affect was constricted but appropriate to context, with no observable signs of mood disturbance or psychotic symptoms. A prior history of simple motor tics and excoriation behaviors was noted, both of which worsened during states of heightened anxiety. No compulsions, obsessions, self-harm, or suicidal behaviors were reported.

A comprehensive neurodevelopmental assessment was conducted for the first time using the Autism Diagnostic Interview-Revised (ADI-R) and the Autism Diagnostic Observation Schedule, Second Edition (ADOS-2). Due to the patient’s severe selective mutism at the time of referral, the ADOS-2 was administered later in the course of treatment, once she had begun to speak with her psychologist. The combined results supported a diagnosis of Autism Spectrum Disorder without disorder of intellectual development and with mild or no impairment of functional language, as defined by the International Classification of Diseases, 11th Revision (ICD-11: 6A02.0) [9]. There had been no previous formal assessment or diagnosis of ASD. A full speech and language evaluation was not feasible during the initial phase; however, based on clinical observation and interaction within the home environment, the patient was subjectively assessed as cognitively competent. Psychometric evaluation using both the child and parent versions of the Screen for Child Anxiety Related Emotional Disorders (SCARED) and the Selective Mutism Questionnaire (SMQ) revealed severe anxiety symptoms with marked elevations in the social and separation anxiety subscales. The diagnoses of Selective Mutism (6B06) and Separation Anxiety Disorder (6B05) were also formalized in line with ICD-11 criteria [9].

Baseline laboratory investigations, including full blood count, liver and renal function tests, thyroid profile, and serum electrolytes, were within normal limits before pharmacological treatment. An ECG was also performed and showed no abnormalities. These assessments were repeated during treatment as clinically indicated, particularly following the introduction of pregabalin, and remained unremarkable.

Management and treatment

Management was tailored to the patient’s high anxiety, sensory sensitivity, and communication barriers, requiring a phased and collaborative approach. The patient was treated in an outpatient setting at a child and adolescent psychiatry clinic. Initial therapeutic efforts focused on establishing a sense of trust and psychological safety. For the first three months, she would only remain in the consultation room if accompanied by her parents. During this period, communication was limited to nonverbal responses, such as nodding. She was extremely resistant to initiating psychotherapy, and all engagement attempts were met with persistent mutism. Nevertheless, her overall anxiety symptoms began to lessen.

In September 2022, she gradually resumed attending school on a partial basis. Upon returning, selective mutism remained fully present in the school setting, with no verbal communication to peers or staff. During this reintegration, she frequently experienced episodes of severe distress at school. These episodes typically occurred during unstructured social situations, such as recess or transitions between classes, and were characterized by sudden crying, trembling, pacing, and requests to leave. In some instances, she clutched her head or clothes, appearing overwhelmed. These symptoms had no identifiable external triggers and occurred approximately four times per week, often prompting school staff to call her parents for early dismissal. Upon returning home, she would quickly regain composure, further reinforcing the school environment as an anxiety-provoking context.

Due to the severity of her anxiety and the inability to initiate psychotherapy effectively, pharmacological treatment was considered early in the intervention. An initial regimen was proposed, including risperidone (0.5 mg nightly), alprazolam extended-release (0.5 mg in the morning), and ethyl loflazepate (2 mg PRN for acute anxiety), guided by off-label clinical practices and supported by pharmacological recommendations for managing severe behavioral and anxiety symptoms in children, as outlined in the Maudsley Prescribing Guidelines [10]. However, the patient persistently refused all medications, in part due to difficulty swallowing pills.

In November, her anxiety significantly worsened, leading to renewed school refusal. After a week and a half of complete absence from school and marked functional deterioration, a shared decision was made with her parents to initiate covert pharmacological treatment. Medication was administered in liquid form by the mother, concealed in cups of water. This approach was adopted as a temporary and carefully monitored measure, aligned with ethical principles and national legal standards, and implemented with fully documented informed consent from both parents. The decision was guided by the principle of acting in the child’s best interest, based on the patient’s lack of insight into her condition and her persistent refusal of care, which posed a serious barrier to therapeutic engagement.

Over the first few months, subtle signs of improvement began to emerge. While her selective mutism remained severe during this period, with no verbal communication outside the home, her overall anxiety gradually decreased. She became more tolerant of attending clinical appointments, albeit non-verbal, and displayed reduced distress during transitions out of the house. By the fourth month, her interactions with her mother became more open, and she began expressing thoughts and feelings more spontaneously at home. These changes culminated in a meaningful shift by the fifth month, when the patient articulated for the first time her awareness of having difficulties and expressed a willingness to receive help. With this newfound insight, medication administration transitioned from covert to voluntary, marking a critical turning point in her clinical trajectory.

Fluoxetine was initiated in November 2022 at 10 mg/day in liquid form and titrated over five months to 60 mg/day. The higher-than-standard dose was used due to partial response at lower doses and was well tolerated with no significant side effects.

Three weeks after initiating fluoxetine, aripiprazole in liquid formulation was added at 5 mg/day to address behavioral rigidity and cognitive inflexibility. The dose was titrated to 10 mg/day. However, during covert administration, the patient developed fatigue and dizziness. Given the long half-life of fluoxetine and the persistence of side effects despite stable dosing, aripiprazole was suspected as the likely cause and was discontinued after approximately two months. Fluoxetine was maintained with continued clinical benefit. Aripiprazole was later reintroduced in April, when the patient began accepting medication voluntarily, and was well tolerated under the direct supervision of the attending child and adolescent psychiatrist.

In April 2023, the patient experienced a notable shift in insight, expressing sadness to her parents about her inability to connect with peers. At that time, she remained completely mute in all environments outside the home, including school. She agreed to start medication voluntarily and presented to emergency services due to distress. Clonazepam 0.5 mg (liquid, nightly) was prescribed in this setting to alleviate acute anxiety. Fluoxetine and aripiprazole were continued during this period, now administered voluntarily. Clonazepam was discontinued after one month due to excessive sedation, dizziness, and poor taste tolerance.

Between May and September 2023, fluoxetine (60 mg/day) and aripiprazole (10 mg/day) were maintained without dosage changes. Selective mutism persisted throughout this period, although gradual improvements were noted in her nonverbal communication and emotional expressiveness at home. She remained mute at school but began tolerating presence in the classroom and participating nonverbally in certain tasks.

In October 2023, pregabalin was initiated at a dose of 25 mg twice daily to target persistent anxiety symptoms and somatic complaints that had not sufficiently responded to fluoxetine and aripiprazole. Although its use is off-label in pediatric populations, the decision was guided by clinical experience and supported by pharmacological recommendations outlined in the Maudsley Prescribing Guidelines in Psychiatry [10]. The rationale for its introduction was based on the continued presence of autonomic hyperarousal and associated functional impairment. The dose was titrated over two weeks to 150 mg/day. At this level, the patient experienced dizziness, nausea, and brief episodes of involuntary saccadic eye movements with diplopia, all of which resolved spontaneously. Repeat blood chemistry and electrolyte assessments remained within normal limits, and neurological examination was unremarkable. Titration was temporarily paused at 100 mg/day and, following symptom resolution, resumed more gradually. The final tolerated regimen was 25 mg in the morning, 75 mg at midday, and 50 mg at night. This schedule led to significant relief of somatic and autonomic anxiety symptoms. At the time of pregabalin initiation, the patient remained on fluoxetine (60 mg/day) and aripiprazole (10 mg/day), both administered voluntarily.

By October 2023, 11 months after starting fluoxetine and shortly after initiating pregabalin titration, the patient began to speak in clinical sessions. Her speech was low in pitch and volume, somewhat monotone, but coherent and articulate. Around the same time, subtle improvements in selective mutism were also observed in the school setting, where she began to whisper brief responses to select staff members during one-on-one interactions, particularly in familiar and low-pressure situations. These early signs of verbal engagement marked the beginning of a broader recovery process.

She began clinical psychotherapy based on cognitive-behavioral therapy (CBT) in April 2023, initially every week and later biweekly, with a month's pause during August. This follow-up continued regularly for more than a year until the moment this report was written. It included psychoeducation on anxiety and ASD, graduated exposure to anxiety-inducing stimuli, and the use of augmentative communication (e.g., yes/no cards). Over time, this progressed to whispered and then full verbal responses. Social skills training and sensory integration strategies were included to target both interpersonal functioning and sensory sensitivities. Parental coaching focused on reducing avoidance accommodations and encouraging gradual exposure. Family involvement remained consistent throughout treatment.

An Individualized Education Plan (IEP) was implemented in collaboration with school staff. Accommodations included written communication, scheduled sensory breaks, and gradual reintegration into verbal classroom participation. Despite frequent absences, she was promoted to the next grade due to good academic understanding and effort. She expressed a desire to remain at her current school due to familiarity with her peers.

From April 2023 onward, the patient demonstrated progressive clinical improvement. By the summer holidays, she began expressing emotions more openly, engaging in conversations with her mother, and showing interest in new foods, clothing, and personal presentation. She chose her outfits, accepted having her fringe trimmed, and initiated public swimming and social play with her brother. She even requested to attend a summer camp, preferring proximity to peers despite not joining all activities.

By September 2023, she was attending school without resistance but continued to struggle with completing full days due to sensory overload. She was now able to verbalize her difficulties clearly, including headaches, exhaustion, and tactile hypersensitivity related to clothes and classroom noise. It was at this point, following the emergence of verbal communication, that the ADOS-2 was administered to further support the diagnostic process for ASD. Full remission of SM was achieved by November 2023. She spoke fluently across all school settings, participated in group discussions, and engaged meaningfully with peers. Though still somewhat reserved, she was now socially functional and academically competent. Her school performance improved, particularly in written work. Exam-related anxiety was managed with PRN mexazolam 1 mg.

At home, she became more emotionally expressive and relaxed. Her dietary and clothing rigidity decreased, and her routines became more flexible. Upon psychometric reassessment, her SCARED total score decreased from 61 to 27, indicating a shift from severe to mild anxiety levels. SMQ ratings normalized across home, school, and social domains, suggesting a full resolution of selective mutism behaviors. While core traits of ASD, such as sensory sensitivities and social reticence, persisted, their impact on daily functioning was considerably reduced.

At the time of reporting, she remained on fluoxetine (60 mg/day), pregabalin (150 mg/day), and aripiprazole (10 mg/day), all taken voluntarily, and continued CBT sessions on a bi-weekly basis. Regular outpatient follow-ups were maintained every four weeks to monitor progress and adjust the treatment plan as needed for six months and then every three months. At the last appointment in December 2024, the patient was functioning well academically and socially. She maintained full verbal communication, attended school daily with little resistance, and reported a good quality of life with improved self-confidence and emotional regulation. At that point, the decision to initiate gradual medication tapering was under active discussion.

**Figure 1:**
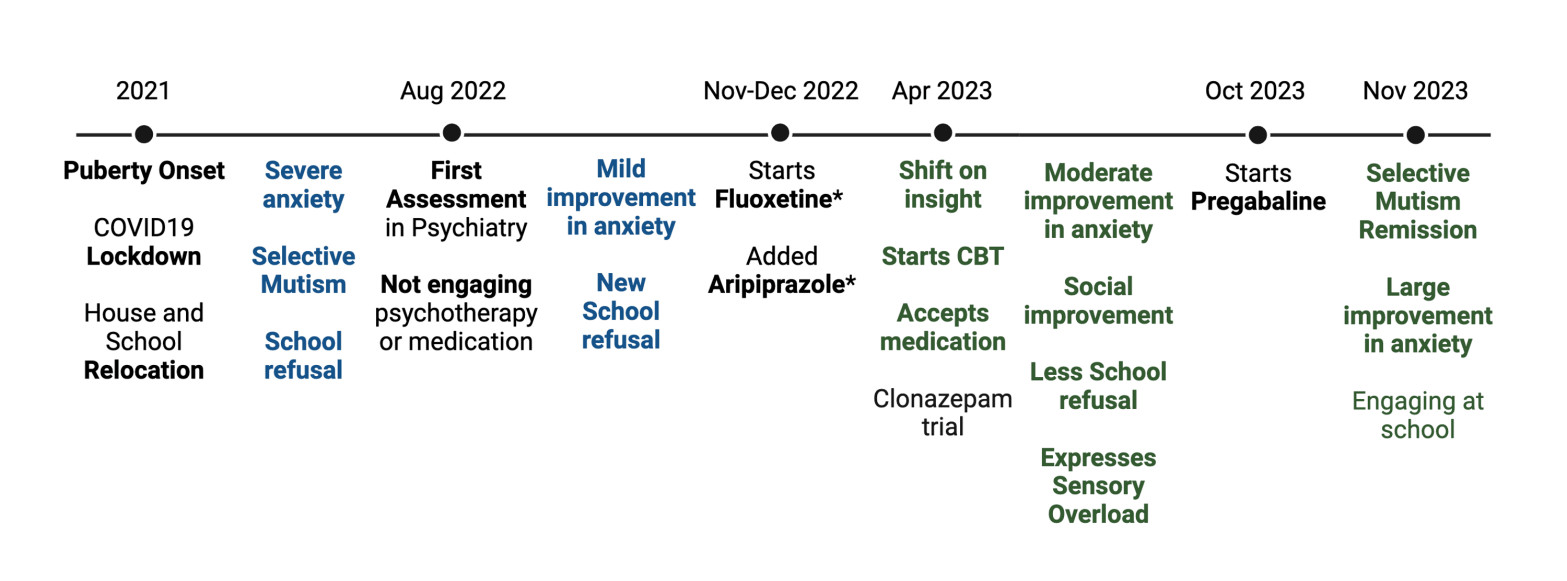
Clinical timeline of symptom onset, interventions, and clinical outcomes. *Medication initially administered covertly with parental consent. In the figure, blue indicates initial symptoms and difficulties, while green represents clinical improvements and outcomes. Additionally, bolded terms indicate key diagnostic events or therapeutic interventions. The image is created by the authors of this study.

For almost two years, the patient progressed from severe functional impairment and mutism to full verbal engagement across settings, improved emotional regulation, and academic reintegration. This outcome was achieved through a combination of CBT, tailored pharmacological support, and intensive family and school collaboration. This clinical course, including symptom onset, therapeutic interventions, and clinical outcomes is summarized in Figure 1.

## Discussion

This study illustrates the complex interplay between ASD, anxiety, and SM during a critical developmental period. Although SM typically presents in early childhood [2], its emergence or exacerbation during puberty - particularly in individuals with ASD - is increasingly recognized as a potential marker of underlying neurodevelopmental vulnerability [4-6]. Puberty is characterized by hormonal, cognitive, and psychosocial changes that may intensify pre-existing anxiety or precipitate maladaptive coping behaviors in predisposed individuals [7].

In youth with ASD, anxiety frequently presents in atypical forms, which can include extreme withdrawal, ritualized behaviors, and context-specific mutism [5]. Distinguishing between ASD-related communication deficits and SM is essential, as the latter is defined by selective, anxiety-driven speech inhibition in specific social contexts, while expressive language remains intact in familiar settings [1,2,8]. This distinction was evident in our case, where the patient spoke freely at home but remained completely mute in public settings, consistent with the phenomenology of SM.

Multimodal treatment approach

While CBT remains the first-line treatment for SM [8,11], pharmacotherapy plays a critical adjunctive role in moderate-to-severe cases, particularly when anxiety symptoms prevent meaningful engagement in therapy [3,12]. Selective serotonin reuptake inhibitors (SSRIs) are the most studied class in pediatric anxiety [11,13], with fluoxetine showing the most robust evidence for efficacy and safety in SM [14,15]. In the present case, a high dose of fluoxetine (60 mg/day) was well tolerated and contributed meaningfully to symptom reduction, in line with prior literature supporting its role in severe cases of SM and comorbid anxiety [11,15].

Pregabalin, although not approved for use in children, has demonstrated efficacy in treating generalized anxiety disorder in adults and may offer therapeutic benefit in adolescents with severe, treatment-resistant anxiety, particularly when characterized by prominent somatic symptoms and autonomic hyperarousal [16]. Its use in our case was guided by the persistence of physiological anxiety symptoms despite SSRI treatment. Initial side effects such as dizziness, nausea, and transient saccadic eye movements required careful dose adjustment and slow titration, highlighting the importance of individualized pharmacological planning. Once stabilized, pregabalin contributed significantly to the reduction of autonomic symptoms and facilitated further therapeutic progress.

The use of aripiprazole, although not central to the treatment of SM, was considered due to prominent behavioral rigidity, ritualistic patterns, and cognitive inflexibility, which are common in ASD and may interfere with adaptation and therapy responsiveness [17]. Its effect on enhancing behavioral flexibility has been supported in prior studies in children with ASD, although close monitoring for sedation and extrapyramidal symptoms remains essential [17].

CBT was progressively implemented once verbal communication and trust were re-established. The therapy followed established principles, incorporating psychoeducation, graded exposure to anxiety-provoking contexts, and the use of augmentative communication strategies to bridge initial mutism [8,11]. In patients with ASD, CBT often requires adaptation, including the use of visual supports, structured routines, and desensitization to sensory triggers [18]. Parental coaching played a central role in reducing accommodation and promoting independence, consistent with evidence supporting family-based approaches [12].

In this case, therapeutic alliance and readiness were fostered slowly, with improvements in engagement and verbal communication emerging only after nearly a year of consistent intervention. This gradual trajectory reflects both the severity of the initial presentation and the need for pacing and flexibility in ASD with comorbid anxiety.

The implementation of an Individualized Education Plan (IEP) in collaboration with the school team was pivotal in supporting re-integration. Accommodations such as allowing written communication, offering sensory breaks, and permitting progressive exposure to verbal demands enabled the patient to re-engage academically. These strategies are consistent with best practices for both SM and ASD in school settings [8,12].

Importantly, the resolution of mutism coincided with broader developmental progress, including improved social engagement, autonomy, and emotional expression. The ability to articulate her internal state and distress, particularly sensory overload, marked a significant clinical milestone and reflected improved self-awareness and emotional regulation.

Ethical considerations

An important ethical and clinical consideration in this case was the initial covert administration of medication. This decision was made due to the severity of the patient’s functional impairment, her refusal to engage in treatment, and her lack of insight into the need for care. Although covert medication is a controversial practice, it may be ethically justifiable in exceptional circumstances involving minors when there is significant risk to well-being. It must be used as a temporary measure, applied with full parental consent, monitored closely, and regularly reviewed. In this case, covert administration allowed for therapeutic engagement to begin and was discontinued once the patient was willing and able to participate in her own care. This approach is consistent with established ethical guidelines for the covert administration of medication in children [19].

Clinical implications

This case underscores that although SM is typically associated with early childhood, its onset or worsening during adolescence, particularly in individuals with ASD, should not be overlooked. Puberty and major life transitions can act as potent triggers for atypical anxiety presentations. These cases require a thorough assessment for ASD and co-occurring anxiety conditions [4-6].

This report contributes to the limited literature on adolescent-onset SM in individuals with ASD. It shows that even severe cases may achieve full remission when approached early with a flexible and individualized multimodal strategy. Importantly, the successful use of pregabalin as an adjunct to SSRI treatment to target autonomic and somatic anxiety symptoms offers a clinically relevant example of how treatment-resistant presentations may benefit from off-label options when standard interventions are insufficient. This case also highlights the ethical and clinical complexities of managing adolescents with profound anxiety and limited treatment insight, including the cautious and temporary use of covert medication with full parental consent.

## Conclusions

Managing SM in adolescents with ASD remains a rare and complex clinical challenge. This case demonstrates that full remission is possible with early, flexible, and multimodal care. A personalized approach combining CBT, school accommodations, and carefully titrated pharmacotherapy, including off-label use of pregabalin, led to marked improvements in emotional, social, and academic functioning.

The temporary, ethically guided use of covert medication enabled therapeutic engagement when standard approaches were not viable. This case contributes valuable insight to the limited literature on adolescent-onset SM in autism, highlighting the need for clinical flexibility and ethical sensitivity. Ongoing collaboration between family and school was crucial to achieving positive outcomes, and medication tapering was being considered at the time of writing.
